# The Freezing Cure

**Published:** 1882-01

**Authors:** J. H. Kellogg


### THE FREEZING CURE.

By means of freezing, parts may be rendered wholly insensible to pain, so that slight surgical operations may be easily performed. When the freezing is long continued, the frozen parts may lose their vitality entirely, which will cause them to slough away. By this means, excrescences, as warts, wens and polypi, fibrous and sebaceous tumors, and even malignant tumors, as cancers, may be successfully removed. Small cancers may sometimes be cured by repeated and long-continued freezing. Their growth may certainly be impeded by this means. A convenient mode of application in cancer of the breast is to suspend from the neck a rubber bag filled with pounded ice, allowing it to lie against the cancerous organ.

Freezing may be accomplished by applying a spray of *ether*, by means of an atomizer, or by a freezing mixture, composed of equal parts of pounded ice and salt, or two parts of snow to one of salt. Mix quickly, put into a gauze bag and apply to the part to be frozen. In three to six minutes, the skin will become white and glistening, when the bag should be removed. Freezing should not be continued longer than six minutes at a time, as the tissues may be harmed, though, usually, no harm results from repeated freezing if proper care is used in thawing the frozen part. It should be kept immersed in cool water, or covered with cloths kept cool by frequent wetting with cold water, until the natural feeling is restored.

Felons may often be cured, especially when they first begin, by freezing two or three times. Lumbago and sciatica, as well as other forms of neuralgia, are sometimes almost instantly relieved by freezing of the skin immediately above the painful part. We have cured some obstinate cases of sciatica by this means after other remedies had failed.—Dr. J. H. Kellogg, in *Physician and Surgeon.*

